# Metagenomic Next-Generation Sequencing of Nasopharyngeal Specimens Collected from Confirmed and Suspect COVID-19 Patients

**DOI:** 10.1128/mBio.01969-20

**Published:** 2020-11-20

**Authors:** Heba H. Mostafa, John A. Fissel, Brian Fanelli, Yehudit Bergman, Victoria Gniazdowski, Manoj Dadlani, Karen C. Carroll, Rita R. Colwell, Patricia J. Simner

**Affiliations:** a Division of Medical Microbiology, Johns Hopkins University School of Medicine, Baltimore, Maryland, USA; b CosmosID, Inc., Rockville, Maryland, USA; c University of Maryland College Park, Institute for Advanced Computer Studies, College Park, Maryland, USA; UAB Clinical Microbiology Laboratory; UNC Health Care System

**Keywords:** COVID-19, nasopharyngeal, SARS-CoV-2, metagenomic next-generation sequencing, metagenomics

## Abstract

Metagenomic next-generation sequencing (mNGS) offers an agnostic approach for emerging pathogen detection directly from clinical specimens. In contrast to targeted methods, mNGS also provides valuable information on the composition of the microbiome and might uncover coinfections that may associate with disease progression and impact prognosis. To evaluate the use of mNGS for detecting severe acute respiratory syndrome coronavirus 2 (SARS-CoV-2) and/or other infecting pathogens, we applied direct Oxford Nanopore long-read third-generation metatranscriptomic and metagenomic sequencing. Nasopharyngeal (NP) swab specimens from 50 patients under investigation for CoV disease 2019 (COVID-19) were sequenced, and the data were analyzed by the CosmosID bioinformatics platform. Further, we characterized coinfections and the microbiome associated with a four-point severity index. SARS-CoV-2 was identified in 77.5% (31/40) of samples positive by RT-PCR, correlating with lower cycle threshold (Ct) values and fewer days from symptom onset. At the time of sampling, possible bacterial or viral coinfections were detected in 12.5% of SARS-CoV-2-positive specimens. A decrease in microbial diversity was observed among COVID-19-confirmed patients (Shannon diversity index, *P* = 0.0082; Chao richness estimate, *P* = 0.0097; Simpson diversity index, *P* = 0.018), and differences in microbial communities were linked to disease severity (*P* = 0.022). Furthermore, statistically significant shifts in the microbiome were identified among SARS-CoV-2-positive and -negative patients, in the latter of whom a higher abundance of *Propionibacteriaceae* (*P* = 0.028) and a reduction in the abundance of Corynebacterium accolens (*P* = 0.025) were observed. Our study corroborates the growing evidence that increased SARS-CoV-2 RNA detection from NP swabs is associated with the early stages rather than the severity of COVID-19. Further, we demonstrate that SARS-CoV-2 causes a significant change in the respiratory microbiome. This work illustrates the utility of mNGS for the detection of SARS-CoV-2, for diagnosing coinfections without viral target enrichment or amplification, and for the analysis of the respiratory microbiome.

## Abstract (precis)

SARS-CoV-2 has presented a rapidly accelerating global public health crisis. The ability to detect and analyze viral RNA from minimally invasive patient specimens is critical to the public health response. Metagenomic next-generation sequencing (mNGS) offers an opportunity to detect SARS-CoV-2 from nasopharyngeal (NP) swabs. This approach also provides information on the composition of the respiratory microbiome and its relationship to coinfections or the presence of other organisms that may impact SARS-CoV-2 disease progression and prognosis. Here, using direct Oxford Nanopore long-read third-generation metatranscriptomic and metagenomic sequencing of NP swab specimens from 50 patients under investigation for COVID-19, we detected SARS-CoV-2 sequences by applying the CosmosID bioinformatics platform. Further, we characterized coinfections and detected a decrease in the diversity of the microbiomes in these patients. Statistically significant shifts in the microbiome were identified among COVID-19-positive and -negative patients, in the latter of whom a higher abundance of *Propionibacteriaceae* and a reduction in the abundance of Corynebacterium accolens were observed. Our study also corroborates the growing evidence that increased SARS-CoV-2 RNA detection from NP swabs is associated with the early stages of disease rather than with severity of disease. This work illustrates the utility of mNGS for the detection and analysis of SARS-CoV-2 from NP swabs without viral target enrichment or amplification and for the analysis of the respiratory microbiome.

## INTRODUCTION

Since the emergence of severe acute respiratory syndrome coronavirus 2 (SARS-CoV-2) in December 2019, cases of coronavirus disease 2019 (COVID-19) have rapidly increased around the world. Research in this area has aggressively expanded, but to date, there have been few metagenomic next-generation sequencing (mNGS) studies of samples from COVID-19 patients. The first study identified SARS-CoV-2 via mNGS of RNA extracted from bronchoalveolar lavage (BAL) samples collected from two patients using an Illumina MiSeq platform (1). Within 6 days, the group was able to identify a novel coronavirus and report a complete genome, demonstrating the utility of mNGS in the early stages of novel pathogen discovery. Other approaches have included random primer metagenomic sequencing (sequence-independent single primer amplification [SISPA]) or metagenomic sequencing with spiked primer enrichment (MSSPE) to identify SARS-CoV-2 using mNGS methods with a limited sample size (2, 3). Most studies have applied an amplicon-based approach to detect and sequence SARS-CoV-2 directly from specimens (1, 2, 4–7).

Perhaps one of the greatest advantages of mNGS is the ability to obtain a snapshot of the patient’s microbiome at a given sampling site to detect coinfections and determine other organisms that may impact patient outcomes. Understanding coinfection is important as it may lead to exacerbation of COVID-19, as was observed with Middle East respiratory syndrome (MERS), and may provide insight into the management of these patients (8). There have been limited reports of coinfection in COVID-19 patients, and thus far, the results have varied, perhaps due to limitations of the different methods used to detect coinfection, poor recovery or detection by standard-of-care methods due to broad-spectrum empirical coverage, lack of testing to understand coinfections, and the diverse geographic regions in which studies were conducted (9–11). Studies from China, the United States, and Europe have found various levels of coinfection within regions as well. These studies report wide ranges of coinfection, from as low as 2% to as high as 80% (12–18). While real-time reverse transcription-PCR (RT-PCR) detection of respiratory pathogens was the most common method used to detect coinfection, there is still a great deal of heterogeneity in the detection methods between these studies. The use of mNGS would not suffer from the same limitations as previous methods and may potentially reveal coinfections that may be missed by the targeted detection methods.

In addition to evidence of true pathogens, there is growing evidence that the microbiome of the respiratory tract can have an impact on the health of patients, but the majority of this evidence focuses on interactions among the bacteria (19). There is growing evidence that it may be possible to predict which patients with respiratory tract infections are more likely to experience more serious disease by analyzing the microbiome (19, 20). There is also evidence that the bacterial burden and composition of the lung microbiome may impact the likelihood that mechanically ventilated critically ill patients will develop acute respiratory distress syndrome (ARDS) (21, 22). Metagenomic analysis of COVID-19 patients provides an opportunity to evaluate whether the microbiome plays a beneficial or deleterious role in patient outcomes (23).

## RESULTS

### Detection of SARS-CoV-2 by mNGS.

**FIG 1:**
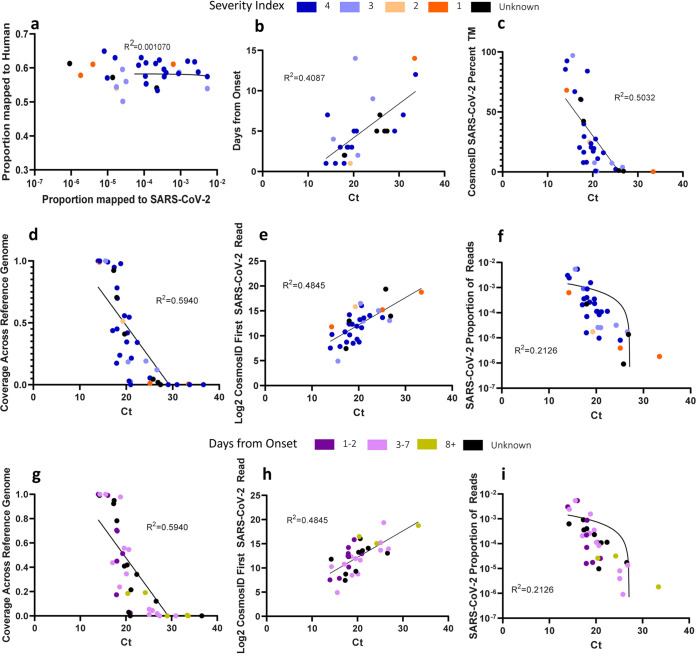
Relationships of metatranscriptomic sequencing sample characteristics to sequencing results. (a) Relationship between the proportion of reads mapped to SARS-CoV-2 and the number of reads matched to human sequences. (b to i) Plots of sequencing results against the Ct values of metatranscriptomic sequencing samples. Panels a to f are color coded by severity index values, and panels g to i are color coded by days from onset of symptoms. The severity index was defined on a scale of 1 to 4, as follows: 4, not admitted; 3, admitted; 2, intensive care unit; and 1, required ventilator. Black dots represent samples with unknown onset or severity index values. (b) Relationship of Ct values determined by LDT-RT-PCR to the number of days from symptom onset. (c) Relationship between Ct values and the proportion of total matches of SARS-CoV-2 by CosmosID. (d, g) Relationship between Ct values and the sequencing coverage across the SARS-CoV-2 strain Hu-1 genome. (e, h) Relationship between Ct values and the number of reads analyzed by CosmosID until the first SARS-CoV-2 read was detected. (f, i) Relationship of Ct values to the proportion of SARS-CoV-2 reads present in the sample. Simple linear regression analysis was performed for each set, and the null hypothesis is rejected for panels b to i (*P* < 0.01), and for each set, *R*^2^ is reported.

First, we demonstrated that SARS-CoV-2 could be detected by direct long-read third-generation metatranscriptomic sequencing from nasopharyngeal (NP) swab specimens of COVID-19 patients collected between 14 and 31 March 2020. SARS-CoV-2 was identified in 31/40 (77.5%) samples that were positive for SARS-CoV-2 by the diagnostic RT-PCR using the online CosmosID bioinformatics program (Table 1). Time to detection of SARS-CoV-2 reads ranged from 1 min (cycle threshold [Ct], 16.0) to 15 h (Ct, 33.4) after the start of the sequencing run, which correlated with the RT-PCR Ct values (Table 1). In the 8 samples where SARS-CoV-2 was unable to be identified, the Ct values ranged from 21.0 to 36.6, with mean and median values of 29.1 and 29.0, respectively (Table 1). We considered that perhaps an abundance of host reads could have masked the SARS-CoV-2 reads in these samples, but there was no relationship between the number of SARS-CoV-2 reads that were detected and human reads detected (Fig. 1a). Lower Ct values were associated with increased sequencing coverage of the SARS-CoV-2 reference genome, a decreased number of total sequencing reads compared to the first SARS-CoV-2 read detection, a greater proportion of SARS-CoV-2 total matches by CosmosID, and a decreasing number of days from the onset of symptoms (Fig. 1b to i). We observed that the most severe cases were spread out along the range of Ct values (Fig. 1d to f). No SARS-CoV-2 reads were identified aligning in any of the 10 samples obtained from patients that were suspected of having SARS-CoV-2 infection but were negative by RT-PCR. A summary of sequencing reads and taxonomic classification of sequencing reads are provided in Tables S1 and S2 in the supplemental material, respectively.

**TABLE 1:** Suspect COVID-19 sample characteristics and sequencing results^*f*^

Patient ID	SARS-CoV-2 RT-PCR Ct value	Severity index^*a*^	No. of days from onset^*b*^	SARS-CoV-2 avg coverage depth (SD)	SARS-CoV-2 genome coverage	% of total CosmosID SARS-CoV-2 matches	SARS-CoV-2 read length, min–max (mean) [median]	Time to SARS-CoV-2 detection (h:min)	Putative pathogen causing coinfection (% genome coverage)^*c*^
COVID-19+^*d*^									
1	16.0	4	1	19.21 (48.40)	99.1	66.8	279–14,387 (1,614.51) [1,260]	0:01	–
2	19.3	2	1	1.83 (3.23)	51.2	24.9	462–9,233 (2,570.95) [1,316]	2:40	Moraxella catarrhalis (6.7)
3	18.6	4	3	0.57 (1.27)	23.8	8.0	504–3,357 (1,611.6) [1,376]	5:30	–
4	29.0	4	5	0 (0)	0.0	No ID	–	–	–
5	18.4	4	3	31.15 (88.21)	97.8	84.0	229–9,676 (1,731.46) [1,409]	0:02	–
6	19.6	4	3	1.80 (3.89)	55.7	23.6	233–4,381 (1,403) [1,161]	0:35	–
7	18.0	4	–	3.83 (8.19)	78.2	39.9	190–5,306 (1,839) [1,647]	0:37	–
8	33.4	1	14	0.01 (0.07)	0.5	0.2	314–314 (314) [314]	15:12	–
9	15.6	3	4	100.26 (237.48)	100.0	96.9	82–14,560 (1,753) [1,466]	0:00	–
10	20.9	3	2	0.07 (0.42)	2.4	0.7	623–1,347 (988) [988]	1:36	–
11	33.6	4	12	0 (0)	0.0	No ID			–
12	20.3	4	–	1.20 (1.85)	41.9	19.5	475–8,505 (2,170.05) [1,611]	0:37	–
13	–	3	10	0 (0)	0.0	No ID	–	–	–
14	21.0	4	–	0 (0)	0.0	No ID	–	–	–
15	21.1	4	–	0.53 (1.19)	21.5	11.1	576–6,104 (2,245.4) [1,778]	1:26	–
16	25.1	4	<7	0.12 (0.47)	5.5	2.2	2,798–2,967 (2,882.50) [2,883]	1:01	–
17	25.1	1	<7	0.02 (0.17)	1.1	No ID	749–749 (749) [749]	2:28	–
18	20.6	4	5	1.64 (2.72)	54.5	27.4	417–6,821 (1,874.82) [1,463]	0:11	–
19	30.9	4	7	0 (0)	0.0	No ID	–	–	–
20	36.6	4	–	0 (0)	0.0	No ID	–	–	–
21	17.1	4	3	1.28 (2.11)	43.7	20.3	349–3,890 (1,505.74) [1,202]	0:12	Human alphaherpesvirus 1 (56.8)
22	18.0	4	2	0.81 (1.13)	45.0	16.4	420–5,331 (1,889) [1,539]	1:13	–
23	17.9	4	1	0.51 (1.66)	17.3	7.8	505–4,245 (1,489.93) [875]	0:07	–
24	24.2	3	9	0.67 (1.68)	19.1	7.3	990–14,306 (3,240.86) [1,247]	1:56	–
25	13.9	4	1	14.14 (23.25)	99.9	85.5	218–16,599 (1,611.98) [1,068]	0:02	–
26	17.4	4	–	5.74 (6.66)	94.9	60.2	312–9,804 (2,111.98) [1,781]	0:02	–
27	17.3	–	–	9.15 (18.71)	92.3	60.5	290–20,914 (1943.81) [1,477]	0:01	–
28	18.1	4	2	6.76 (18.51)	69.8	29.5	318–5,930 (1,838.85) [1,627]	0:08	–
29	20.6	4	–	0.03 (0.17)	2.9	0.8	1,017–1,017 (1,017) [1,017]	10:25	Haemophilus influenzae (23.3)
30	14.2	–	–	11.73 (21.78)	99.0	68.1	307–10,590 (2,131.49) [1,793]	0:11	–
31	20.1	1	5	1.92 (5.10)	34.5	17.2	395–6,014 (1,816.36) [1,534]	0:02	–
32	14.3	4	7	29.77 (61.23)	99.9	92.5	299–17,295 (1,866.7) [1,551]	0:08	–
33	25.8	4	7	0.05 (0.21)	4.4	1.1	3,034–3,034 (3,034) [3,034]	12:42	Haemophilus influenzae (5.2)
34	26.6	–	–	0.16 (0.44)	12.0	3.9	725–3,091 (2,216.75) [2,526]	0:51	–
35	26.8	3	5	0.02 (0.14)	1.9	0.6	663–663 (663) [663]	4:01	–
36	22.4	–	–	1.19 (2.50)	34.1	15.9	822–5,207 (2034.61) [1,572]	1:35	–
37	20.4	4	14	0.28 (0.67)	18.5	7.7	796–2,696 (1,676.67) [1,567]	6:33	–
38	18.0	3	2	4.37 (7.29)	70.5	42.3	307–17,619 (1,835.43) [1,241]	0:21	–
39	19.7	–	–	1.93 (4.25)	41.0	20.5	341–6,919 (1,828.77) [1,526]	0:05	–
40	27.4	4	<7	0 (0)	0.0	No ID	–	–	Human metapneumovirus (99.3)

COVID-19–^*e*^									
41	NA	4	–	0 (0)	0	No ID	–	–	–
42	NA	4	3	0 (0)	0	No ID	–	–	–
43	NA	–	–	0 (0)	0	No ID	–	–	–
44	NA	1	8	0 (0)	0	No ID	–	–	*–*
45	NA	4	4	0 (0)	0	No ID	–	–	–
46	NA	4	1	0 (0)	0	No ID	–	–	–
47	NA	4	6	0 (0)	0	No ID	–	–	–
48	NA	4	3	0 (0)	0	No ID	–	–	Moraxella catarrhalis (5.6)
49	NA	4	4	0 (0)	0	No ID	–	–	–
50	NA	4	–	0 (0)	0	No ID	–	–	–

a The severity index was defined on a scale of 1 to 4, as follows: 4, not admitted; 3, admitted; 2, intensive care unit; and 1, required ventilator.

b Days from onset is defined as the number of days from the initial onset of symptoms until the time of specimen collection.

c Putative pathogens causing coinfections are defined as known pathogens, with bacteria having above 50% relative abundance and not considered microbiota.

d Suspected COVID-19 specimens that were found to be positive for SARS-CoV-2 by RT-PCR.

e Suspected COVID-19 specimens that were found to negative for SARS-CoV-2 by RT-PCR.

f Ct, cycle threshold; SD, standard deviation; –, unknown information; No ID, samples that were not identified by CosmosID; NA, not applicable.

10.1128/mBio.01969-20.1 TABLE S1 Summary of sequencing reads. Download Table S1, XLSX file, 0.03 MB. Copyright © 2020 Mostafa et al. 2020 Mostafa et al. This content is distributed under the terms of the Creative Commons Attribution 4.0 International license.

10.1128/mBio.01969-20.2 TABLE S2 Taxonomic classification matrices for metagenomic and metatranscriptomic data. Download Table S2, XLSX file, 0.3 MB. Copyright © 2020 Mostafa et al. 2020 Mostafa et al. This content is distributed under the terms of the Creative Commons Attribution 4.0 International license.

### Identifying coinfections.

Next, we examined the samples for possible coinfections (Table 1). Of the 40 COVID-19-positive samples, 5 (12.5%) revealed organisms of clinical relevance detected in high abundance (>50% relative to normal levels in microbiota). These included Haemophilus influenzae (*n* = 2; 5%), Moraxella catarrhalis (*n* = 1; 2.5%), human metapneumovirus (hMPV) (*n* = 1; 2.5%), and human alphaherpesvirus 1 (*n* = 1; 2.5%) (Table 1). In our COVID-19-negative samples, Moraxella catarrhalis (*n* = 1; 10%) was identified. Unfortunately, standard-of-care testing was not performed to detect these pathogens. No fungal or protist coinfections were detected.

### Evaluating the respiratory microbiome.

**FIG 2:**
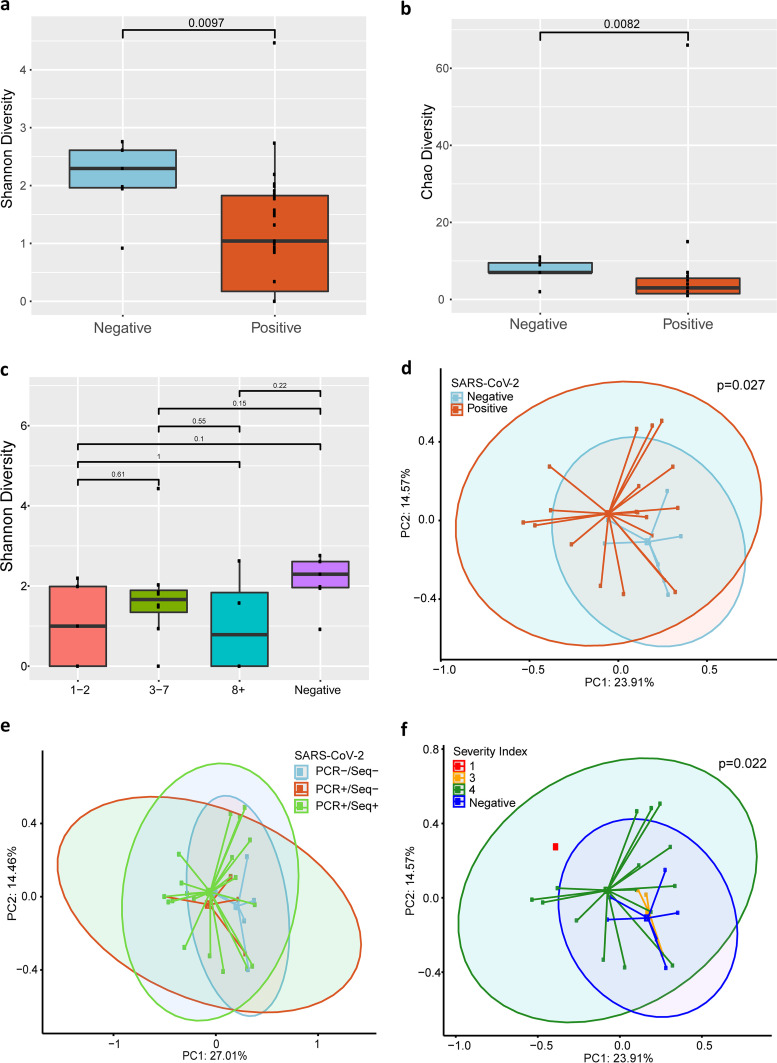
Bacterial diversity analysis of metagenomic sequencing results. (a, b, c) Alpha diversity analysis of metagenomic sequencing results. SARS-CoV-2 positivity was determined by LDT-RT-PCR. (a, b) Shannon diversity plot and Chao diversity plot of SARS-CoV-2-negative and -positive samples at the species level. (c) Shannon diversity plot of SARS-CoV-2-negative samples and SARS-CoV-2-positive samples at different periods post-onset of symptoms. (d, e, f) Beta diversity principal-coordinate analysis of metagenomic sequencing results at the species level. (d) Bray-Curtis analysis of bacterial community composition diversity between SARS-CoV-2-negative and SARS-CoV-2-positive samples. (e) Bray-Curtis analysis of bacterial community composition diversity grouped by PCR and sequencing positivity. (f) Bray-Curtis analysis of bacterial diversity in different disease severity groups. (a, b) Wilcoxon rank sum tests were performed between positive and negative SARS-CoV-2 groups for the Shannon diversity index (*P* = 0.0097) and Chao diversity (*P* = 0.0082). (c) Wilcoxon rank sum tests were performed between disease onset groups (no significance). (d, f) PERMANOVA tests were performed on Bray-Curtis distance matrices for SARS-CoV-2-positive and -negative groups (*P* = 0.027) (d) and groups of disease severity (*P* = 0.022) (f). (e) Pairwise PERMANOVA tests were performed on Bray-Curtis distance matrices between groups defined by SARS-CoV-2 positivity by RT-PCR and sequencing. RT-PCR+/sequencing+ (PCR+/Seq+) versus RT-PCR–/sequencing– (PCR–/Seq–) (*P* = 0.007); PC1 and -2, principal components 1 and 2. The severity index was defined on a scale of 1 to 4, as follows: 4, not admitted; 3, admitted; 2, intensive care unit; and 1, required ventilator.

Beyond determining coinfections, we analyzed the metagenomic profiles of these patients in order to uncover potential shifts in the microbiome that could impact patient outcomes. Specimens obtained from SARS-CoV-2-positive patients did have a significant reduction in the diversity of their bacterial communities at the species level as measured by the Shannon diversity index (*P* = 0.0082), Chao richness estimate (*P* = 0.0097) (Fig. 2a and b), and Simpson diversity index (*P* = 0.018). We did not see significant differences at the genus and family levels. Given that we did see a decrease in diversity in the positive samples, we were interested in determining if there was decreasing diversity at lower Ct values. However, among these samples, we did not observe any relationship between Ct values and diversity (data not shown) using the same analyses described above. Further, we also assessed whether there was a relationship with days from symptom onset and found that there was no difference using these analyses (Fig. 2c).

In order to further compare the microbial community compositions for these patients at the family, genus, and species levels, we utilized Bray-Curtis principal-coordinate analysis (PCoA). When comparing the communities in SARS-CoV-2-positive and SARS-CoV-2-negative patients, we saw a significant difference (*P* = 0.027) between the two groups at the species level (Fig. 2d) but not at the genus and family levels. We also analyzed whether there was a difference between samples grouped by RT-PCR and sequencing positivity (Fig. 2e). There was no difference between RT-PCR-positive/sequencing-negative (RT-PCR+/sequencing–) samples and RT-PCR+/sequencing+ samples (*P* = 0.372) or between RT-PCR+/sequencing– samples and RT-PCR–/sequencing– samples (*P* = 0.064) at the family, genus, or species level. There was a significant difference between the RT-PCR+/sequencing+ samples and RT-PCR–/sequencing– samples (*P* = 0.007) at the species level. In addition, we also observed a difference at the species level when we compared patients’ samples grouped by severity index (*P* = 0.022) (Fig. 2f). As was the case in the previous PCoA analysis, there was no significant difference at the genus and family levels.

**FIG 3:**
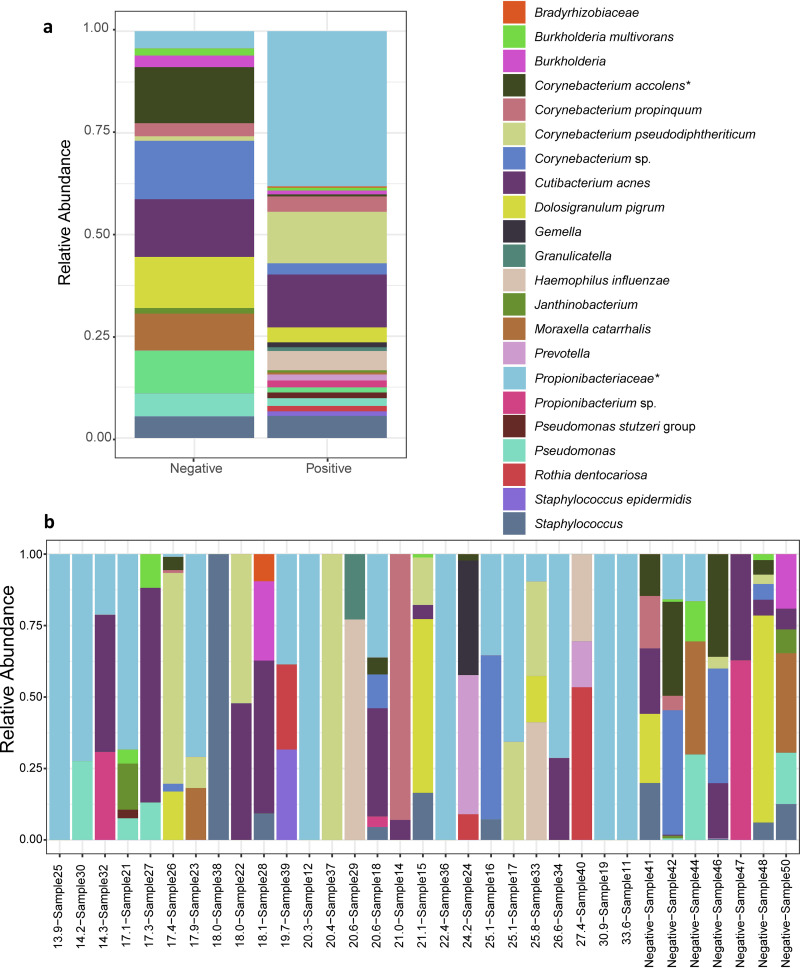
Changes in bacterial microbiome composition in COVID-19 patients. (a, b) Relative abundances of bacteria at the species level in COVID-19-positive and COVID-19-negative samples. (a) Overall relative abundances of bacterial species in COVID-19-positive and COVID-19-negative patients. (b) Relative abundances of bacterial species in individual COVID-19-positive and COVID-19-negative patients, with samples ordered by Ct value determined by LDT-RT-PCR. A Wilcoxon rank sum test was performed on overall relative abundance values for *Propionibacteriaceae* (*, *P* = 0.028) and for Corynebacterium accolens (*, *P* = 0.025).

Finally, we visualized the microbial community composition at the species level by comparing the overall relative abundances of species detected in SARS-CoV-2-positive and SARS-CoV-2-negative samples. The family *Propionibacteriaceae* revealed the greatest difference in abundance between these two groups, with *Propionibacteriaceae* proportionately more abundant in SARS-CoV-2 patients by ca. 30%, representing the most abundant organism group detected in these samples (*P* = 0.028) (Fig. 3a). Additionally, there was a significant decrease in the incidence of Corynebacterium accolens in COVID-19-positive patients (*P* = 0.025) (Fig. 3a). With respect to individual samples, we observed that, in many cases, a single organism comprised the vast majority of sequencing reads detected among the SARS-CoV-2-positive patients (Fig. 3b).

## DISCUSSION

In this study, we demonstrated that it is possible to detect SARS-CoV-2 in NP swab specimens by direct long-read metatranscriptomic sequencing. Due to the relatively low cycle threshold (Ct) values (i.e., high viral burdens) observed in these samples, it was possible to obtain sufficient sequencing reads without amplification or enrichment of viral targets during preparation of the cDNA library. These steps are often necessary to obtain sufficient sequencing depth or to overcome background reads, particularly in low-titer samples (24–26). However, eliminating the amplification or enrichment step prior to sequencing introduces less bias in sequences that are ultimately generated by this method. Further, forgoing PCR amplification preserves the long sequencing reads that maximize the utility of this platform. Our study also corroborates growing evidence that the viral load in the nasopharynx is highest early in the disease course and wanes as disease progresses (27, 28).

From our metagenomic analysis, we were able to observe a reduction in the microbial diversity of SARS-CoV-2-positive patients, which may be driven in part by several samples overwhelmingly dominated by a single species. Interestingly, single-species predominance was observed in samples throughout the range of Ct values. Using PCoA analysis, we also detected a significant difference in the microbial communities when grouped by severity index. However, it should be noted that there were larger numbers of samples in the less severe groups.

A recent study of critically ill patients found that an increased abundance of *Enterobacterales* in respiratory samples increased the risk of developing ARDS (21). This observation suggests that gut-colonizing bacteria may have a deleterious impact on outcomes when present in the respiratory tract. However, in our study, we did not observe increased gut-colonizing organisms. *Propionibacteriaceae* were more abundant in our SARS-CoV-2-positive patients. However, *Propionibacterium* and *Cutibacterium* (formerly *Propionibacterium*) species may represent sampling contaminants since they are a component of normal skin microbiota (29–31). Previous reports have associated *Propionibacteriaceae* with the respiratory tract in cystic fibrosis patients, but thus far, no linkage with disease severity has been established (29, 32). We also observed a significant reduction in the relative abundance of Corynebacterium accolens in our COVID-19 patients. This organism is considered a commensal organism, and there is evidence that it has a negative association with colonization by Streptococcus pneumoniae (30, 31, 33). Further studies would be required to conclude the role of these associations in patients with COVID-19.

At the time of sampling, we did observe several clinically relevant organisms that may be potential causes of coinfection. Haemophilus influenzae (*n* = 2) and Moraxella catarrhalis (*n* = 1) were identified in COVID-19-positive samples with high relative abundances. These organisms frequently colonize the respiratory tract preceding infection, have been associated with exacerbations of chronic bronchitis and pneumonia (34, 35), and have previously been detected in COVID-19 patients (11, 36). Interestingly, there is also evidence that viral infections of the respiratory tract can increase the ability of H. influenzae to establish infection (34, 37, 38). We identified the viral pathogen human metapneumovirus (hMPV) in a COVID-19-positive sample. Although typically associated with the common cold, this virus has also been associated with exacerbations of pulmonary disease, particularly in the very young and the elderly (34, 39). In addition, we identified human alphaherpesvirus 1 (HSV1) in one patient, which likely represents reactivation in the setting of COVID-19. In our COVID-19-positive samples, we observed that the SARS-CoV-2 reads were overwhelmingly abundant and that this virus frequently was the only virus identified in the metatranscriptomic sequencing results.

There are limitations to our study. First is the inability to draw larger conclusions regarding the composition of the microbiome and associations with COVID-19, including its impact on the severity of disease due to the relatively low number of samples, particularly from the more severe categories of the severity index. Having a randomized sample selection approach was beneficial to demonstrate that it is possible to detect SARS-CoV-2 from samples at various Ct values seen in our patient population. However, this ultimately led to relatively few patients with severe disease being included in our study. More broadly, a larger sample size would be necessary for future studies in order draw more definitive conclusions about microbial diversity in COVID-19 patients. In order to assess the utility of direct long-read metatranscriptomic sequencing as a potential sentinel method for assessing the continued sensitivity of RT-PCR assays, future studies should also include greater numbers of samples from suspected COVID-19 patients that are RT-PCR negative. Our study did not detect SARS-CoV-2 in any of the 10 samples from patients suspected of having COVID-19 that were negative by RT-PCR despite both our study groups having no difference in mean number of days from symptom onset, which suggests that this observation was not due to the reduced clinical sensitivity reported for NP swabs from later stages of disease. Single-nucleotide polymorphism analysis to study genomic diversity for the majority of our samples was not pursued due to insufficient depth or incomplete viral genome coverage.

In summary, this study serves as a proof of concept that it is possible to detect SARS-CoV-2 and characterize simultaneous coinfections and the respiratory microbiome of patients under investigation for COVID-19 using direct long-read metatranscriptomic and metagenomic sequencing.

## MATERIALS AND METHODS

### Ethics statement.

This study was approved by the Johns Hopkins University institutional review board, with a waiver of informed consent.

### Sample collection and severity index assignment.

In this study, 50 randomly selected nasopharyngeal (NP) swab specimens were obtained from 40 COVID-19 patients and 10 patients suspected of having COVID-19 but negative by a diagnostic laboratory-developed test (LDT), RT-PCR (U.S. Food and Drug Administration Emergency Use Authorization pending), on gene targets E and S (Table 2) (40). Remnant NP swabs were collected for the study at the completion of standard-of-care testing prior to disposal. We assigned a four-point severity index value to the samples based on the following criteria: 4, the patient was not admitted to the hospital; 3, the patient was admitted to the hospital but not the intensive care unit (ICU); 2, the patient was admitted to the ICU; and 1, the patient was placed on a ventilator.

**TABLE 2:** Patient demographics and clinical characteristics

Demographic information or clinical characteristic	Value(s)
Total no. of patients	50
SARS-CoV-2 positive	40
SARS-CoV-2 negative	10

Median age in yr (IQR)^*d*^ [range]	50.5 (36–63) [18–78]

No. (%) of sex:	
Male	26 (52)
Female	24 (48)

No. (%) of race^*a*^:	
Total	38
African American	17 (44.7)
Asian	2 (5.3)
White	19 (50)

No. (%) with the following comorbidities:	
Total	19 (38)
Cardiovascular disease	
Hypertension	14 (28)
Metabolic disease	
Obesity	2 (4)
Diabetes mellitus	3 (6)

No. (%) of days from onset of symptoms^*b*^	
1–2	9 (18)
3–7	19 (38)
>7	7 (14)
Unknown	15 (30)

No. (%) of patients with severity index^*c*^:	
1	4 (8)
2	1 (2)
3	6 (12)
4	33 (66)
Unknown	6 (12)

a Self-reported in prespecified fixed categories.

b Number of days from onset of symptoms until specimen collection.

c The severity index was defined by a 4-point scale: 4, not admitted to hospital; 3, admitted to hospital; 2, admitted to ICU; and 1, required ventilator.

d IQR, interquartile range.

### Nucleic acid extraction.

Automated total nucleic acid extraction was performed using the NucliSENS easyMag (bioMérieux, Marcy-l'Étoile, France) software, version 2.1.0.1. The input volume was 500 μl, and the elution volume was 50 μl. Extraction was performed by following the manufacturer’s protocol. Extracts were stored at –80°C prior to mNGS sequencing.

### mNGS GridION sequencing.

Long-read metagenomic sequencing was performed using the Nanopore GridION X5 (Oxford, England) sequencing instrument. Each Nanopore sequencing library was prepared using total nucleic acid extract from NP swabs. Two different libraries were generated for each specimen using two different kits. A direct cDNA sequencing kit (SQK-DCS 109; Oxford Nanopore Technologies) was used for sequencing poly(A)^+^ RNA full-length transcripts to allow for sequencing SARS-CoV-2 in a nonbiased way. In addition, we used a PCR barcoding kit (SQK-PBK004; Oxford Nanopore Technologies) for capturing DNA targets for untargeted metagenomic analysis and to complement the metatranscriptomic analysis. For cDNA libraries, complementary cDNA strand synthesis, as well as strand switching, was performed using kit-supplied reagents and oligonucleotides. This was followed by RNA strand degradation and synthesis of complementary strands and then ligation of sequencing adaptors. Multiplexing was performed using the Native barcoding expansion kit (EXP-NBD104).

For PCR barcoding kit libraries, briefly, total nucleic acid was fragmented using the Covaris g-TUBE. Repair of sheared ends was performed using the NEBNext end repair/dA-tailing, followed by ligation of adaptors containing primer binding sites for sample amplification as well as barcoding. Primers used, in addition to incorporating barcodes, added 5′ tags to allow for sample attachment to one-dimensional rapid sequencing adaptors.

Specimens were sequenced using R9.4.1 flow cells (FLO-MIN106). Five samples were multiplexed per flow cell, except for libraries for samples 1 to 5, which were prepared with the Direct cDNA sequencing kit and sequenced one sample per flow cell. Samples 1 to 10 have only metatranscriptomic data, as there was insufficient remaining specimens for subsequent metagenomic sequencing. MinKNOW software was used to collect and base call the sequencing data.

### CosmosID analysis and bioinformatics pipeline.

Raw nanopore fastq files that passed filter based on nanopore default parameters were concatenated into one file per sample, with all reads in order of sequencing start time. Concatenated, unassembled sequencing reads were directly analyzed by the CosmosID bioinformatics platform (CosmosID Inc., Rockville, MD) as described elsewhere (41–44) for multi-kingdom microbiome analysis and quantification of organism relative abundance. Briefly, the system utilizes curated genome databases and a high-performance data-mining algorithm that rapidly disambiguates hundreds of millions of metagenomic sequence reads into the discrete microorganisms engendering particular sequences. Four variables are generated for each organism detected: unique match frequency, unique match percentage, total match percentage, and relative abundance, as previously defined (45). The CosmosID % Total Matches statistic was used as an approximation of the percent coverage of the sample against SARS-CoV-2 strain Hu-1.

Sequencing reads were mapped against SARS-CoV-2 strain Hu-1 using the tool Minimap2, with default parameters. Output sam/bam files were sorted and indexed using SAMtools, and summary statistics were generated using Qualimap (46–48).

To determine the detection, depth, and coverage of SARS-CoV-2 strain Hu-1 at different times, multiple cumulative read files were generated per sample. Depending on the number of reads in a sample, subsamples were created using the first 1,000, 2,000, 4,000, and 8,000 reads, doubling until a maximum of 256,000 reads was reached. These files were used for both CosmosID and Minimap analysis. The number of the first read identified was the first read mapped to SARS-CoV-2 strain Hu-1 via the CosmosID Metagenomics software. Approximations of the amounts of reads belonging to various kingdoms were determined by the number of reads in each sample that mapped to the CosmosID database for each kingdom.

Relative abundance stacked bars were generated from the family-, genus-, and species-level relative abundance matrices from CosmosID taxonomic analysis. Bars are separated based on the comparative cohort and were visualized using the R package ggplot2 (49).

Putative pathogens causing coinfections are defined as organisms of high abundance having >50% relative abundance compared to that in the normal microbiota (i.e., organisms expected to be found in the respiratory tracts of healthy individuals). Orthogonal validation of coinfection calls were completed by running raw nanopore output files against reference genomes of interest using minimap-2, with default parameters, in the same way as was used in the mapping to SARS-CoV-2 strain Hu-1. Mapping statistics and coverage plots were generated from Minimap’s sam/bam files using Qualimap’s bamqc function.

### Statistical analysis.

Alpha diversity boxplots were calculated from the family-, genus-, and species-level abundance score matrices employing the CosmosID taxonomic analysis. Chao, Simpson, and Shannon alpha diversity metrics were calculated in R using the R package vegan. Wilcoxon rank sum tests were performed between positive and negative COVID-19 groups using the R package ggsignif. Boxplots with overlaid significance in *P* value format were generated using the R package ggplot2 (49–51).

Beta diversity principal-coordinate analyses (PCoA) were calculated from the family-, genus-, and species-level relative abundance matrices from CosmosID taxonomic analysis. Bray-Curtis and Jaccard diversities were calculated in R using the R package vegan with the function vegdist, and PCoA tables were generated using vegan’s function pcoa. Permutational multivariate analysis of variance (PERMANOVA) tests for each distance matrix were generated using vegan’s function adonis2. Group pairwise PERMANOVAs were generated using the pairwise Adonis function pairwise.adonis2. Plots were visualized using the R package ggpubr (50, 52, 53).
